# Retinotopic mapping of the primary visual cortex – a challenge for MEG imaging of the human cortex

**DOI:** 10.1111/j.1460-9568.2011.07777.x

**Published:** 2011-08

**Authors:** Gavin Perry, Peyman Adjamian, Ngoc J Thai, Ian E Holliday, Arjan Hillebrand, Gareth R Barnes

**Affiliations:** 1 The Wellcome Trust Laboratory for MEG Studies, School of Life and Health Sciences, Aston UniversityBirmingham, UK; 2 CUBRIC, School of Psychology, Cardiff UniversityPark Place, Cardiff CF10 3AT, UK; 3 MRC Institute of Hearing Research, University ParkNottingham, UK; 4 Clinical Research & Imaging Centre, University of BristolBristol, UK; 5 Department of Clinical Neurophysiology, VU University Medical CenterAmsterdam, The Netherlands; 6 Wellcome Trust Centre for Neuroimaging, Institute of Neurology, University College LondonLondon, WC1N 3BG UK

**Keywords:** gamma oscillations, inverse, localization, magnetoencephalography, primary visual cortex

## Abstract

Magnetoencephalography (MEG) can be used to reconstruct neuronal activity with high spatial and temporal resolution. However, this reconstruction problem is ill-posed, and requires the use of prior constraints in order to produce a unique solution. At present there are a multitude of inversion algorithms, each employing different assumptions, but one major problem when comparing the accuracy of these different approaches is that often the true underlying electrical state of the brain is unknown. In this study, we explore one paradigm, retinotopic mapping in the primary visual cortex (V1), for which the ground truth is known to a reasonable degree of accuracy, enabling the comparison of MEG source reconstructions with the true electrical state of the brain. Specifically, we attempted to localize, using a beanforming method, the induced responses in the visual cortex generated by a high contrast, retinotopically varying stimulus. Although well described in primate studies, it has been an open question whether the induced gamma power in humans due to high contrast gratings derives from V1 rather than the prestriate cortex (V2). We show that the beanformer source estimate in the gamma and theta bands does vary in a manner consistent with the known retinotopy of V1. However, these peak locations, although retinotopically organized, did not accurately localize to the cortical surface. We considered possible causes for this discrepancy and suggest that improved MEG/magnetic resonance imaging co-registration and the use of more accurate source models that take into account the spatial extent and shape of the active cortex may, in future, improve the accuracy of the source reconstructions.

## Introduction

The problem of reconstructing electrical activity in the brain based on magnetic measurements recorded extracranially using magnetoencephalography (MEG) is ill-posed. In order to gain a unique solution, prior constraints must be imposed. At present there are a multitude of inversion algorithms each employing different assumptions, all verifiable in simulation under the appropriate conditions (Baillet *et al.*, 2001; Darvas *et al.*, 2004). One major problem is that, for most experimental data, the correct solution (i.e. the true electrical state of the brain) is unknown. In this study, we explore one paradigm, retinotopic mapping in the visual cortex, for which the ground truth is known to a reasonable degree of accuracy, but which potentially presents some interesting and difficult challenges for MEG inverse algorithms.

It is known from both lesion (Horton & Hoyt, 1991) and functional magnetic resonance imaging (fMRI) (Sereno *et al.*, 1995; Engel *et al.*, 1997; Tootell *et al.*, 1998) studies in humans that the primary visual cortex (V1) is organized retinotopically. Using MEG, a transient evoked response following stimulus onset has been found to localize to V1 (Aine *et al.*, 1996; Portin *et al.*, 1999; Supek *et al.*, 1999; Moradi *et al.*, 2003). Recent studies (Gramfort *et al.*, 2007; Yoshioka *et al.*, 2008; Hagler *et al.*, 2009) show that, when certain fMRI/anatomical constraints are imposed on the inversion, the recovery of the retinotopic organization of these responses is possible. Although the temporally modulated stimuli that many of these studies use are known to give strong visual evoked fields/potentials (Fylan *et al.*, 1997; Hermann, 2001; Fawcett *et al.*, 2004), we also know that such stimuli excite many visual areas (see the literature on retinotopic mapping, e.g. Engel *et al.*, 1997; Sereno *et al.*, 1995). For MEG this becomes a problem as the modeling of these multiple distinct areas of active cortex is inherently non-linear and unstable (hence the need for functional and anatomical constraints).

In this study we attempted to simplify the problem (of multiple sources) by localizing the gamma-band induced response due to a high contrast grating stimulus. Static high contrast patterns are known in primates to give rise to sustained increases in the local field potential coherence between local neuronal populations in V1 (Rols *et al.*, 2001; Leopold & Logothetis, 2003; Henrie & Shapley, 2005). These sustained coherence changes have been shown to follow a retinotopic organization in V1 but are largely absent in the prestriate cortex (V2) (Rols *et al.*, 2001). Thus, such visual stimuli give rise to a retinotopically mapped response that appears to be constrained to a single cortical area.

These stimuli have also been shown in humans to give rise to an MEG/electroencephalography measurable mean field signal in the gamma range, which can be localized to the occipital cortex (Adjamian *et al.*, 2004b; Hall *et al.*, 2005b; Hoogenboom *et al.*, 2006; Swettenham *et al.*, 2009; Muthukumaraswamy *et al.*, 2010). Based on these findings, we sought to use the known retinotopic properties of gamma oscillations to test the localization accuracy of a particular non-linear beanforming technique (Robinson & Vrba, 1999).

## Materials and methods

**Fig. 1:**
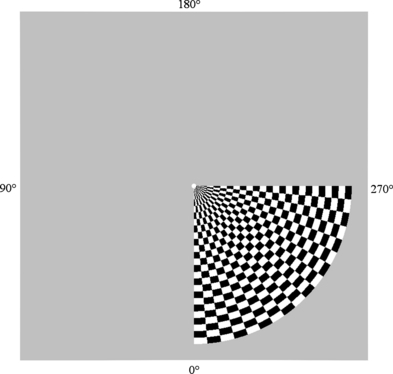
The experimental stimulus.

Data were recorded using a 275-channel whole-head MEG system (CTF Systems Inc., Port Coquitlam, Canada) at a sampling rate of 600 Hz, from 10 participants all of whom gave their written informed consent to participate (the study conformed with The Code of Ethics of the World Medical Association). For each participant, data were acquired in a single trial in which they passively viewed a rotating ‘wedge’ stimulus (Fig. 1) while fixating on a central spot. The gratings were generated using a Cambridge Research Systems VSG 2/5 grating generator and displayed on an Eizo Flexscan T560i, gamma-corrected, color monitor at a frame rate of 100 Hz. Participants viewed the screen through a small window in the wall of the magnetically shielded room.

The experimental stimulus was a ‘wedge’-shaped checkerboard of 90° arc extending from fixation to 3.75° of visual angle, presented on a gray background (see Fig. 1). The stimulus contained 24 checks along its radius (equivalent to a spatial frequency of 3.2 cycles/degree) and 18 checks along its arc (equivalent to a spatial frequency of approximately 5.89 cycles/degree at the outer edge, and decreasing as the pattern approached fixation by a factor of 1/*d*, where *d* is the distance to the apex). The Michelson contrast within the stimulus was 80%. During viewing the wedge rotated around fixation in a clockwise direction at a rate of 16°/s, meaning that the wedge took 22.5 s to complete a single 360° rotation. Importantly, there was no temporal modulation on the checkerboard itself and the stimulus could alternatively be considered as a static checkerboard over which a transparent window moves. Individual participants viewed between 23 and 30 rotations of the stimulus in a single session. Eye movements were not tracked, but a small red fixation point was present at the apex, which flickered black at random intervals, and participants were instructed to fixate this point and count the number of flickers, in order to ensure that their eyes remained fixated at the apex of the wedge.

For the purpose of the synthetic aperture magnetometry (SAM) beanformer analysis, the data recorded over each rotation of the stimulus wedge were divided into 23 overlapping time windows, each of 5.625 s in duration (this was the time that it took the full arc of the wedge to cross over any individual point in the visual field). Data covariance was calculated separately for each of these time windows for each frequency band tested (see below), and the source power was reconstructed for a grid of dipolar sources with a resolution of 2 × 2 × 2 mm, using a multisphere model of volume conduction (Huang *et al.*, 1999), i.e. data covariance for any stimulus location was calculated from the concatenation of data across all rotations of the stimulus from one of the above 23 time windows. The source power was reconstructed separately for activity in the 30–60 Hz (gamma), 15–25 Hz (beta), 8–12 Hz (alpha) and 4–7 Hz (theta) frequency ranges. No artifact rejection was performed.

Three types of beanformer metric were computed: pseudo-*T*, pseudo-*F* and pseudo-*Z*. The pseudo relates to the fact that the estimates of variance in these statistics are based on an estimate of the sensor noise level and not between-epoch variability. In brief, the pseudo-*T* gives the difference (between two conditions) in estimated source power relative to the projected noise level; the pseudo-*F* gives the ratio of source power (in two conditions); and the pseudo-*Z* is an absolute measure of source power divided by the estimate of projected noise at that location (for details see Robinson & Vrba, 1999; Vrba & Robinson, 2001). Noise was estimated using the lowest eigenvalue of the data pooled across conditions (see Hillebrand & Barnes, 2005). For *T* and *F* metrics, the ‘active’ time window was compared with an ‘opposing’ time window starting 11.25 s (i.e. half a cycle) later in the sequence (activity generated by the stimulus at a given position was compared with activity due to the stimulus at the opposite angular location in the visual field). For the *Z* metric, active power alone was used in the computation.

Activity was localized by finding the source location with the largest power in the posterior part of the cortex within each of the time windows, so that the data were finally represented as a series of 23 points corresponding to the location of the peak source for each stimulus location. Sources with a magnitude of half the projected white noise level (pseudo-*T*, 0.5) or less were not considered for peak localization, meaning that, where no source exceeded 0.5 in a time window for a given beanformer metric, no peak was found for that time window (this threshold was chosen during exploratory analyses as the maximal value at which we could still recover peaks at all positions for at least two participants). Thus, in some of the results presented not all stimulus positions are mapped.

During recording, the head position was continually localized by measuring the locations of three emitting coils attached to fiducial points on the participants’ scalp. For the two participants (AH and SW) who were selected for detailed analysis, head movements did not exceed 5 mm in any individual recording session. Prior to data collection, a three-dimensional digitizer (Polhemus Isotrak; Kaiser Aerospace Inc.) was used to digitize each participant's scalp, brow and nose surface (typically at least 300 points), and this was used to co-register an anatomical magnetic resonance image of each participant with the MEG sensors using surface-matching software (Adjamian *et al.*, 2004a) in order to provide an accurate measurement of head location, both for the estimation of the lead field matrix and to allow the beanformer results to be plotted relative to each individual's cortical surface. The mean error of fit between the scalp and magnetic resonance imaging (MRI) surface points for each participant was typically in the region of 0.5 mm.

The Freesurfer software package (http://surfer.nmr.mgh.harvard.edu/) was used to segment the gray/white matter surface from the anatomical image (Dale *et al.*, 1999). Peak locations for all time windows were overlaid in a single figure on a corresponding anatomical ‘slice’ that consisted of all points from the segmented surface falling within 2 mm of the mean peak location along the direction perpendicular to the ‘slice’. All figures are plotted in coordinates of the participants’ anatomical MRI.

## Results

We initially focussed our analysis on gamma oscillatory activity, by computing pseudo-*T* comparisons in the 30–60 Hz range for all 10 participants. This meant that, for each participant, 23 source reconstructions were created corresponding to gamma oscillatory power induced by stimulation in each of one of 23 angular locations around fixation. Sources were localized by finding the largest source (pseudo-*T* > 0.5) in the posterior part of the cortex within each time window, so that data were finally represented as a series of 23 points corresponding to the location of the peak source for each stimulus location.

**Fig. 2:**
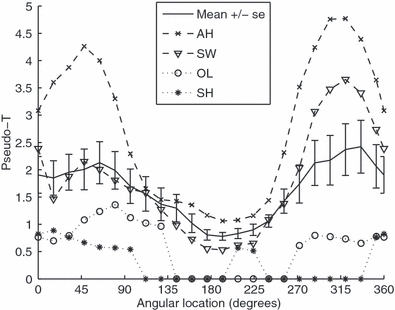
Plot of peak pseudo-*T* values in the 30–60 Hz frequency range for each angular location for four subjects (AH, SW, OL and SH), as well as the group mean with bars showing the standard error of the mean. Peak voxels with pseudo-*T* < 0.5 were not included in the analysis. Locations are defined so that 0° and 360° represent the vertical position in the lower half of the visual field. AH and SW were the two subjects selected for further analysis, whereas OL and SH are examples of subjects with a weak response.

Figure 2 shows a plot of mean peak pseudo-*T* value across the 10 participants as a function of wedge angle. Also shown are the two participants, AH and SW, who were selected for detailed analysis (see below) and the two participants with the weakest responses. It is clear that responses were much better across the group for lower visual field stimuli.

**Fig. 3:**
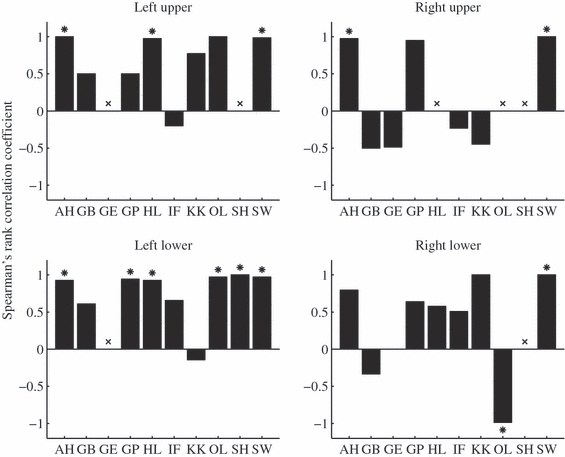
Plots of the Spearman's rank correlation coefficient between the angular location of the stimulus and the angular position of the corresponding source for each participant. Each panel shows the correlation separately for one of the four visual quadrants. ^×^Quadrants for which less than three peaks were recovered and so no correlation coefficient could be calculated. *Correlations that were significant at the *P* < 0.05 level.

If these peak sources map retinotopically then, as the stimulus rotates clockwise, the sources should similarly rotate clockwise (if the sources are localized in V1) or anticlockwise (if the sources are localized in V2). In order to test this, for each participant we calculated an ‘origin’ by taking the mean peak source location in the coronal plane (i.e disregarding the source's position along the anterior–posterior axis), and then for each time window we calculated the angle between the line through the ‘origin’ and the peak source in the coronal plane and the (negative) vertical axis. In order to test whether this source angle varied consistently with the angular location of the stimulus, we calculated the Spearman's rank correlation coefficient between the two quantities separately for each visual quadrant in each participant, and the results are shown in Fig. 3. Seven quadrants (from four participants) were excluded from this analysis due to having less than three peaks recovered within the quadrant. Of the remaining 33 quadrants, 13 quadrants (from six participants) had a significant (*P*< 0.05) correlation.

The signs of these correlation coefficients indicate the direction of rotation of the sources (either clockwise or anticlockwise as the stimulus moved clockwise) within each quadrant. We reasoned that if the sources were being generated consistently by a single area of the retinotopic cortex within and across participants then the direction of rotation should also be consistent across quadrants, and should be positive (i.e. moving clockwise) if the sources were generated in V1 and negative (i.e. anticlockwise) if the sources were generated in V2 (due to the opposite field signs of the two areas). Of the 33 quadrants for which a correlation coefficient could be calculated, 24 had positive coefficients and eight had negative coefficients (the remaining quadrant had a correlation coefficient of zero, and hence was not included in the statistical analysis). Taking as our null hypothesis that, in the event of no consistent retinotopic mapping, the correlation coefficients should be equally likely to be positive or negative for a given quadrant, a binomial test revealed that for our data this null hypothesis could be rejected (*P*= 0.007; exact binomial test, two-tailed).

To demonstrate that this effect was not dependent on using quadrants for which the correlation was not significant, we repeated the analysis using only the 13 quadrants with correlations that were different from zero at the *α*= 0.05 significance level. Of these 13, only one quadrant had a negative coefficient (see Fig. 3). Again, this is significantly different from the null hypothesis (*P*= 0.003; exact binomial test, two-tailed). Hence we are able to conclude that, for visual quadrants in which the rotation of the source locations was correlated with the rotation of the stimulus, the direction of rotation was consistent across quadrants at a level significantly greater than chance. Moreover, as there were significantly more positive correlations (and hence activity moved in a clockwise direction as the stimulus moved clockwise) the data are consistent with V1 (and not V2) being the origin of the measured gamma activity, congruent with primate neurophysiology (Rols *et al.*, 2001).

Participants were selected for more detailed analysis based on the following criteria. Firstly, that they had a pseudo-*T* peak with a magnitude larger than 0.5 in the posterior part of the cortex for all time windows (meaning that the full 360° rotation of the stimulus could be mapped), and secondly, that they had correlation coefficients significantly greater than zero in all four quadrants (*α*= 0.05, one-tailed). Of the original 10 participants, two (AH and SW) met both of these criteria, and it is these two participants for whom further analysis was carried out.

**Fig. 4:**
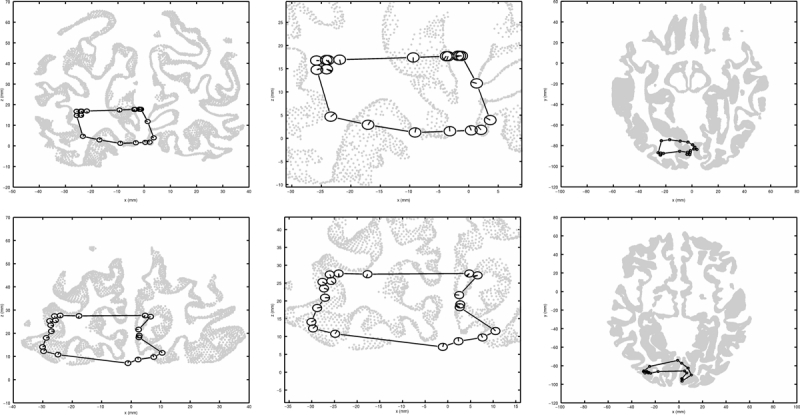
Locations of the strongest cortical source (peak pseudo-*T*) in the 30–60 Hz frequency range as the stimulus rotated around the visual field, for subject AH (top row) and subject SW (bottom row). The central panel in each row shows a close-up of the sources from a coronal view, whereas the smaller panels show the data against a full cross-section of the cortical gray/white matter boundary from coronal (left) and axial (right) views. Each source location is represented in the figure by a ‘clock face’ centered on the location of the peak pseudo-*T* value, with the position of the line(s) in each ‘clock’ indicating the angular location(s) within the visual field at which the stimulus produced that peak.

The locations of the sources found for AH and SW are plotted in Fig. 4 in coronal and axial views, overlayed onto corresponding slices of the cortical gray/white matter boundary for both participants. The figure reveals that the relative locations of the sources in both participants are consistent with V1 retinotopy, with the up–down, left–right mirror reversals of the retinotopic map known to occur in the visual cortex. Interestingly, it is notable that for SW there is a clear separation between sources produced by the left and right visual fields, consistent with the two hemifields being represented in the two hemispheres of the cortex (although this separation is less apparent for AH). It is also notable that the relatively continuous mapping around the horizontal meridian in both visual fields strongly suggests that the source locations are not consistent with activation of extrastriate visual regions, where the horizontal meridia are bisected by earlier visual areas and where we would therefore expect sharp discontinuities in the source location as the stimulus passed through the horizontal meridian. This provides further evidence that the sources were generated in area V1.

However, it is also clear from Fig. 4 that the locations of the source peaks relative to the cortical surface do not conform to what would be expected from activity generated from V1. Anatomically, area V1 is known to occupy the area in and around the calcarine sulcus, and thus electrical sources generated by V1 activity should fall around that sulcus. The actual source locations appear shifted to the left, and spread out over a much wider area than would be expected from sources originating in V1.

**Fig. 5:**
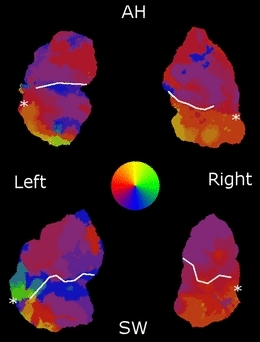
Flattened surfaces extracted from the medial occipital cortex in the right and left hemispheres of participants AH and SW. Each point on the surface is colored according to preference for the angular location of the stimulus, as derived from volumetric images of pseudo-*T* magnitude. The white line and asterisk on each surface indicate the approximate locations of the fundus of the calcarine sulcus and the occipital pole, respectively.

As an alternative approach to visualizing retinotopy, we treated each participant's pseudo-*T* data as a series of three-dimensional volumetric images (one for each time window) and found the time window (and hence angular location) with the greatest pseudo-*T* magnitude for each voxel across the set of images. This enabled us to look at the distributed pattern of location preference across all voxels rather than just at the position of the peak source. The volumetric data were then projected onto the Freesurfer-segmented gray/white matter boundary, and plotted as surface maps of location preference, as is commonly done in fMRI studies of retinotopy. Figure 5 shows these results plotted on flattened surfaces extracted from the medial occipital cortex. The results show that, for both participants, data in the right hemisphere are consistent with the known retinotopic properties of V1, with preferred locations being in the left visual hemifield, and activity shifting from dorsal to ventral regions of the occipital cortex as the stimulus moves from the lower to the upper visual field. In contrast, in the left hemisphere in both participants, the preferred locations across much of the surface are in the left visual hemifield – inconsistent with known retinotopy. Findings for both hemispheres can be explained with reference to the peak source locations shown in Fig. 4 where the peak source locations for the left visual field appear close to the medial surface of the occipital cortex in the right hemisphere, but are shifted over to the lateral surface of the left hemisphere for the right visual field. Thus, sources from the left visual hemifield are closest to the medial surfaces of both the left and right hemispheres, and this explains why the left hemisphere maps appear to show a preference for the left visual field. This suggests that, when using the SAM beanformer, distributed voxel-wise measures of retinotopy do not differ radically from those that can be inferred solely by measuring the peak source location.

Pseudo-*F* results were also calculated for both participants (not shown). Although the precise locations of the sources found differed by as much as 2 cm from the pseudo-*T* results, the broad pattern of the data was similar to that shown in Fig. 4, with the source locations following a roughly retinotopic mapping in the source space, but failing to be localized to the cortical surface. Consistent with the results shown in Fig. 4, the strongest sources were found when the stimulus was in the lower right of the visual field (i.e. sources in the upper left of the visual cortex) for both participants.

**Fig. 6:**
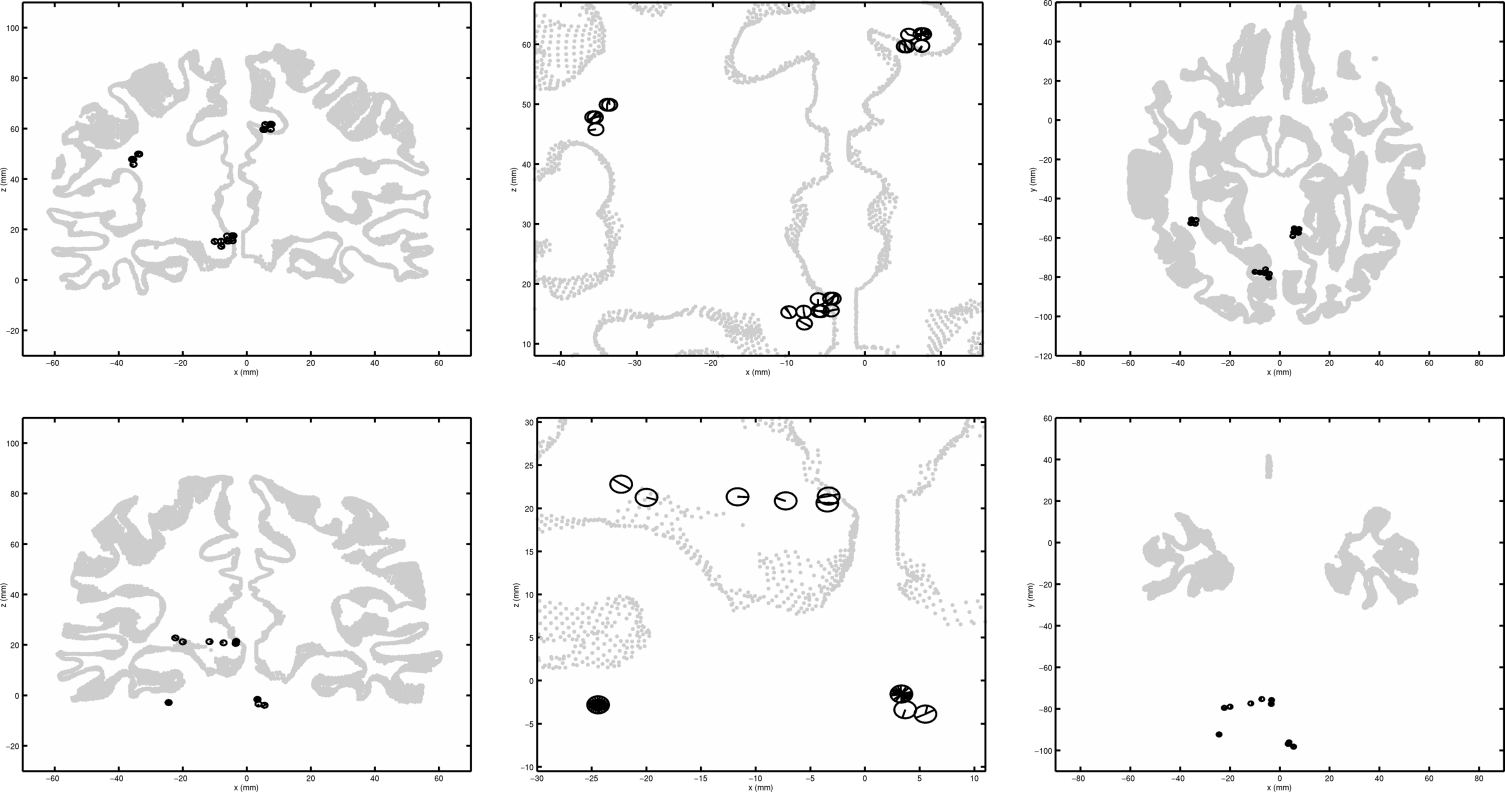
Locations of cortical sources (peak pseudo-*Z*) in the 30–60 Hz frequency range as the stimulus rotated around the visual field, for subject AH (top row) and subject SW (bottom row). The central panel in each row shows a close-up of the sources from a coronal view, whereas the smaller panels show the data against a full cross-section of the cortical gray/white matter boundary from coronal (left) and axial (right) views. Each source location is represented in the figure by a ‘clock face’ centered on the location of the peak pseudo-*Z* value, with the position of the line(s) in each ‘clock’ indicating the angular location(s) within the visual field at which the stimulus produced that peak.

Figure 6 shows the results for the pseudo-*Z* statistic, i.e. absolute, rather than relative, power is mapped as a function of location. In contrast to the previous results, it is clear that pseudo-*Z* results do not show any specific retinotopic organization, and in fact most reconstructed sources did not appear to show any significant changes of location as stimulus location changed (see Discussion). In order to demonstrate the absence of a retinotopic mapping for any source peaks, all positive peaks (rather than just the largest peak) with a pseudo-*Z* value larger than 0.5 for each time window are plotted in Fig. 6.

**Fig. 7:**
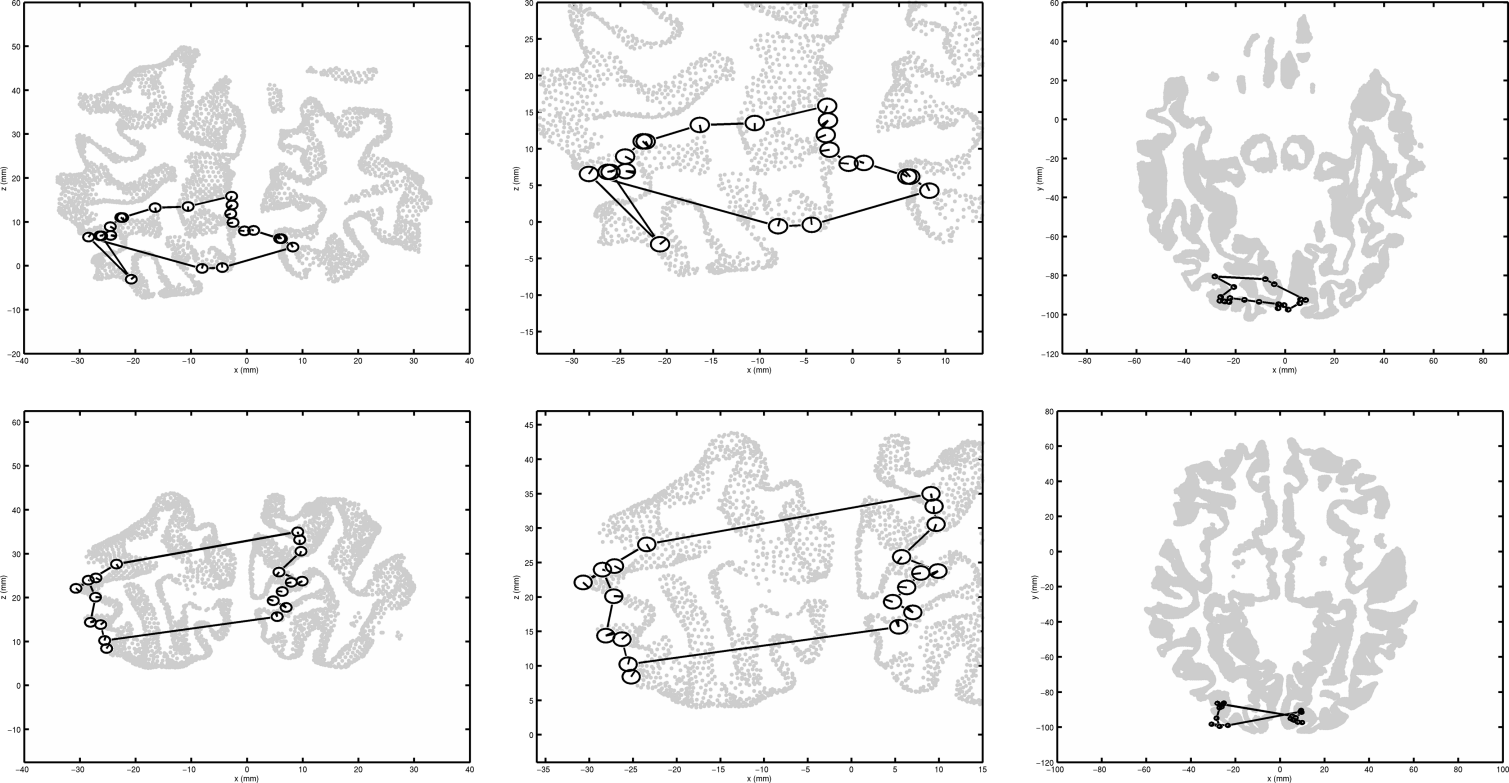
Locations of the strongest cortical source (peak pseudo-*T*) in the 4–7 Hz frequency range as the stimulus rotated around the visual field, for subject AH (top row) and subject SW (bottom row). The central panel in each row shows a close-up of the sources from a coronal view, whereas the smaller panels show the data against a full cross-section of the cortical gray/white matter boundary from coronal (left) and axial (right) views. Each source location is represented in the figure by a ‘clock face’ centered on the location of the peak pseudo-*T* value, with the position of the line(s) in each ‘clock’ indicating the angular location(s) within the visual field at which the stimulus produced that peak.

Whereas no consistent results were found in the 8–12 and 15–25 Hz frequency ranges, we did find that, in addition to the gamma activity, participants showed large power differences in the theta (4–7 Hz) band. The locations of the peak pseudo-*T* statistic for each time window are plotted in Fig. 7. Although the precise locations of the peaks differ from those found in the gamma frequency range, the overall pattern is the same, with the peaks mapping retinotopically, but with the absolute locations of the peaks failing to map onto the cortical surface.

**Fig. 8:**
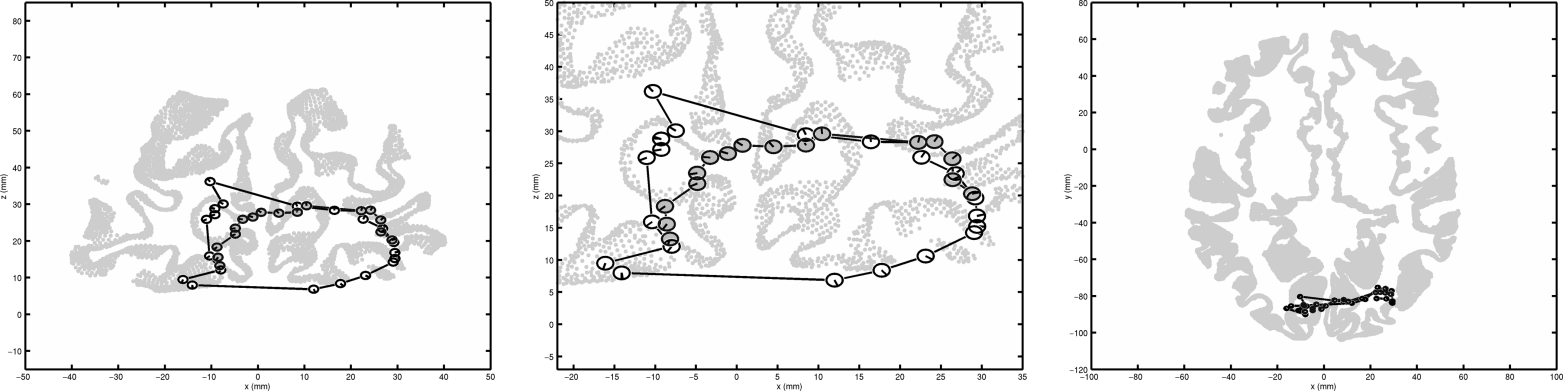
Locations of the strongest cortical source (peak pseudo-*T*) in the 30–60 Hz frequency range as the stimulus rotated around the visual field, measured in the second session with subject SW. The central panel shows a close-up of the sources from a coronal view, whereas the smaller panels show the data against a full cross-section of the cortical gray/white matter boundary from coronal (left) and axial (right) views. White ‘clocks’ show results from the trial with stimulus radius of 3.75°, and gray ‘clocks’ show results from the trial with stimulus radius of 1.25°.

There are two main factors that could account for the discrepancy between the actual and expected source localizations – co-registration and modeling error. In order to test the consistency of the results across recording sessions (co-registration error), SW was retested in a second recording session. Data were recorded from two trials in this second session; the first trial was identical to the one used in the original recording session, whereas in the second trial the stimulus radius was reduced in size to 1.25°. The reduction in stimulus size was meant to reduce the amount of active cortex and hence mitigate effects of modeling error (a simple dipole model is used with synthetic aperture magnetometry, which is most appropriate as a model for a small piece of active cortex). Figure 8 shows the locations of the peaks in activation for both trials overlaid on the cortical surface, revealing that a retinotopic mapping is present for both stimulus sizes, albeit only in those locations where discernible peaks were present for the smaller stimulus.

These results show that, firstly, the locations of the sources relative to the cortical surface are similar across trials in the second session, but are clearly shifted when compared with Fig. 4. This suggests that part of the deviation from the true source locations is due to a factor that varies between recording sessions, most likely the accuracy of co-registration. However, it also appears that the variation is largely in the form of a translation along the left–right axis, and hence is unlikely to explain all of the difference between the observed results and those expected based on V1 retinotopy. Secondly, the similarity between the localization of sources across the two trials indicates that changes in the size of the stimulus do not alter the localization of the sources, at least within the range of sizes tested (see also Discussion below).

## Discussion

In this study we have suggested that gamma oscillatory activity in V1 might be a particularly useful but challenging benchmark for testing the accuracy of MEG source localization methods. We can divide our findings into two main sections.

### Physiology

Physiologically we have shown that human stimulus-induced oscillations in both the 30–60 and 4–7 Hz frequency range map in a deterministic manner consistent with V1 retinotopy. To our knowledge, this is the first time that the externally measured gamma-band signature presented in a number of non-invasive MEG studies (Adjamian *et al.*, 2004b; Hall *et al.*, 2005a,b; Hoogenboom *et al.*, 2006; Muthukumaraswamy *et al.*, 2009, 2010; Swettenham *et al.*, 2009) has been attributed unequivocally to V1 rather than V2, due to the trajectory of the peaks. This is consistent with invasive primate recordings (Rols *et al.*, 2001).

In addition, within individuals we have shown that lower visual field stimuli give rise to significantly larger modulations in gamma-band activity than upper visual field stimuli, consistent with previous findings (Portin *et al.*, 1999). This could be due to the larger amount of the cortex devoted to lower visual field stimuli (Van Essen *et al.*, 1984), or possibly (but less likely) to decreased MEG sensitivity to the more inferior portions of the cortex (cf. Portin *et al.*, 1999).

Crucially, retinotopy was found only when the cortical power was contrasted across retinal locations using pseudo-*T* and pseudo-*F* statistics. When absolute cortical power was measured, using the pseudo-*Z* statistic, gamma activity did not appear to map retinotopically. This suggests that the most powerful gamma sources present during the current task were not modulated by retinal location, and that only stimulus contrasts reveal retinotopically-organized activity in V1. One possible confound is that the mean-gray luminance monitor on which the stimulus was presented could have given rise to a large standing gamma oscillation in the visual cortex. This is difficult to control for, as we did not record a monitor-off condition. Such monitor-induced gamma activation would be consistent with the large pseudo-*Z* peak observed in the approximate center of the retinotopic locus for AH, but not for SW. This does not detract from the retinoptopic movement of the peaks but may help to explain the location errors, i.e. our localization bias could be due, in part, to a large number of constantly active uncorrelated gamma generators perturbing the beanformer accuracy (see also Hillebrand and Barnes, 2011).

A limitation to the stimuli used here is clearly the large inter-participant variability in the magnitude of the induced electrical changes (see also Muthukumaraswamy *et al.*, 2010). The main advantage, however, is that the source model is relatively simple compared with temporally modulated stimuli.

### Methodology

Although MEG imaging of V1 might at first seem straightforward, it presents serious challenges for MEG inverse methods. Firstly, the calcarine sulcus is a highly convoluted area of the brain with much of its area hidden within the folds of the cortical surface (Van Essen *et al.*, 1984). This makes it particularly susceptible to modeling errors and cancellation effects of activity in differently oriented patches of the active cortex. Secondly, previous observations suggest that visual gamma responses are highly variable between individuals, both in terms of response magnitude and peak frequency (Muthukumaraswamy *et al.*, 2010). Hence, retinotopic mapping of V1 presents some difficult challenges for MEG inverse methods.

Although this is, to our knowledge, the first study to attempt to retinotopically map visually-induced gamma oscillations, a number of previous MEG studies have attempted retinotopic mapping of V1 using visually evoked responses (Sharon *et al.*, 2007; Yoshioka *et al.*, 2008; Hagler *et al.*, 2009; Brookes *et al.*, 2010). In some cases a high degree of concordance between the location of the fMRI blood oxygenation level-dependent (BOLD) response in V1 and the source location of evoked responses has been found (Moradi *et al.*, 2003; Sharon *et al.*, 2007). However, other evidence suggests that visually evoked responses have multiple, distributed sources originating in the extrastriate as well as striate cortex (Aine *et al.*, 1996; Supek *et al.*, 1999; Hall *et al.*, 2005a,b; Poghosyan & Ioannides, 2007; Koelewijn *et al.*, 2011). This is problematic for MEG inverse methods, as the modeling of these multiple distinct areas of the active cortex is inherently non-linear and unstable. Moreover, until the source distribution of visually evoked activity is better understood, it is difficult to assess whether or not any individual study has accurately reconstructed the true source state of the cortex. In contrast, the physiological evidence suggests that induced gamma power has its origin primarily in V1 (Rols *et al.*, 2001), and hence it seems reasonable to assume a single source origin in the striate cortex, simplifying the ground truth against which the accuracy of any particular attempt at source localization must be compared.

In this study we have verified that MEG inversions based on the beanformer give physiologically reasonable trajectories of source peaks consistent with V1 retinotopy. However, when co-registered with structural MRI data it was found that the gamma sources did not localize to the area around the calcarine sulcus (where V1 is located), or even necessarily to any part of the cortical surface. This was surprising as we had deliberately chosen a small stimulus, sacrificing the signal-to-noise ratio for a simple electrical source structure.

One possibility is that estimates of the data covariance matrices were inaccurate, which would have affected the accuracy of the source reconstructions (Brookes *et al.*, 2008). One way to militate against such an effect would be to apply regularization, which effectively reduces the sensitivity of the beanformer to noise in the covariance matrix at the expense of some spatial resolution; in the limit (of large regularization) tending to a single dipole fit (Hillebrand & Barnes, 2003). We empirically tested a number of levels of regularization (from 10 to 50 000), but all values resulted in a degraded retinotopy (as quantified by the Spearman's rank correlation coefficient) over the default value of zero used here. Alternatively, a ‘common filter’ approach, where the beanformer weights are based on data from several conditions and the beanformer output is based on a contrast between source power estimates for separate conditions, would increase the amount of data and therefore the accuracy of the data covariance estimates. However, with this approach the beanformer output is reduced when the sources for different conditions produce different field patterns. Further studies should determine the optimum strategy for the computation of beanformer weights in cases where MEG signals are produced by closely spaced (uncorrelated) sources. Another possibility is that the source localization is in fact accurate, but the co-registration between MRI and SAM data is inaccurate. Certainly the method used to co-register the two kinds of data is susceptible to a number of sources of error, such as MRI distortions, inaccuracies in the digitization of the scalp surface, movement of the reference coils during MEG data acquisition or failure of the co-registration algorithm to find an optimal match between the MRI and the scalp surface (Schwartz *et al.*, 1996; Brinkmann *et al.*, 1998; Kozinska *et al.*, 2001; Whalen *et al.*, 2008). That said, we used a standard method and obtained reasonably accurate digitized head shapes [typically more than 300 points, consistent with the findings of Whalen *et al.* (2008) that co-registration accuracy asymptotes around 200–300 points]. Moreover, the second recording session with SW showed a clear shift (∼2 cm) in source locations relative to the original session, suggesting that there is some variation in co-registration between sessions. The influences of misregistration of the fiducial coils will increase monotonically as one moves away from the center of the head, but even so we were surprised at the magnitude of this error in the occipital cortex.

Given that co-registration errors could have been present and may have accounted for the translation in the coronal plane, the fact that the location of the power peaks would not map to the calcarine sulcus even with a compensatory translation suggests that our approach also suffered from a degree of modeling error. We know from previous studies that, as the signal-to-noise ratio increases, the accuracy of beanformer source models becomes increasingly important (Vrba, 2002; Hillebrand & Barnes, 2003). Here, we attempted to model the activity over an extended area of cortex using a single dipole source. Our results would therefore imply that gamma coherence in the human cortex is extended over a much larger distance than would be expected from primate studies (in which gamma sources appear to be locally coherent in the range of a few millimetres) (Steriade *et al.*, 1996; Gail *et al.*, 2000). This is unlikely as human data seem to conform to the animal models (Bullock *et al.*, 1995), and mismodeling of the spatial extent of activation might therefore not fully account for our observations. Despite this, a more accurate alternative model of the cortical activity was constructed, namely a series of dipoles spread over a region of the cortex. We ran a series of analyses on the current data using seven dipoles arranged hexagonally along a single plane, with the distance between dipoles varying from 1 cm to 0 mm (being equivalent to a single dipole). However, for both participants there was no improvement in accuracy with additional dipoles, and a general decrease in pseudo-*T* values as the dipoles became more distantly spread.

This result is consistent with recent simulation work (Hillebrand & Barnes, 2011), which suggests that curvature, not the two-dimensional spatial extent, is a much more critical factor when attempting to model the magnetic field due to an extended region of the cortex [see Hillebrand & Barnes (2002) for an analogous problem of localization error introduced when a single dipole is used to model a curved area of the cortex]. Hence, we believe that greater localization may be achieved in future studies if detailed information about the three-dimensional shape of the cortical source, such as may be derived from MRI, is used to constrain the shape of a multidipolar source model (Cottereau *et al.*, 2007). Of course, such complex models would be counterproductive if the co-registation were not accurate (Hillebrand & Barnes, 2003, 2011); however, in this case a less spatially selective method (such as a minimum norm solution) might suffice. Indeed, the use of such anatomical information has proved successful in differentiating V1 and V2 sources in electroencephalography (Ales *et al.*, 2010).

Based on fMRI measures of the cortical magnification in humans (Dougherty *et al.*, 2003; Qiu *et al.*, 2006), we estimate that our full-sized stimulus would have activated a region of cortex in excess of 300 mm^2^. An alternative explanation for our results might be that, although there is no coherence *per se* between local populations of V1 neurons, the larger the area of stimulation (and hence the number of coherent sub-populations) the more likely the possibility of distant generators that are coherent simply due to chance. This would violate the main assumption behind the beanformer technique, namely that distant sources are uncorrelated (Barnes & Hillebrand, 2003; Hillebrand *et al.*, 2005), and reduce its accuracy. In order to try to mitigate this effect we did reduce the stimulus size, although we estimate that the reduced stimulus still activated at least 150 mm^2^, a large area relative to the coherence domains found by Rols *et al.* (2001). Interestingly, even with the reduced stimulus, the peaks mapped to very similar locations. Moreover, it is noteworthy that even the maximum stimulus eccentricity used in this study was 3.75°, whereas typical fMRI studies use much larger stimuli (e.g. Sereno *et al.*, 1995; Engel *et al.*, 1997; Tootell *et al.*, 1998; Dougherty *et al.*, 2003).

In summary, our results demonstrate that the localization of gamma activity in V1 provides a difficult challenge for MEG inverse methods. We chose a paradigm to give the simplest MEG source model possible, at the expense of the signal-to-noise ratio. We did see retinotopic movement of source location with stimulus position. However, even in our best participants we, surprisingly, were not able to reconstruct a trajectory consistent with V1 anatomy. Importantly, the use of this well-described physiological phenomenon (retinotopy of gamma oscillations) has given us a metric of the distortion (without which we might have been content with our findings). We would suggest that highly convoluted cortical areas such as V1 pose particular challenges for MEG, and so we consider the ability to construct retinotopic maps to be of critical importance in developing MEG imaging techniques.

## Abbreviations

fMRI: functional magnetic resonance imaging
MEG: magnetoencephalography
MRI: magnetic resonance imaging
SAM: synthetic aperture magnetometry
V1: primary visual cortex
V2: prestriate cortex
